# Metagenomic Analysis of the Microbiota from the Crop of an Invasive Snail Reveals a Rich Reservoir of Novel Genes

**DOI:** 10.1371/journal.pone.0048505

**Published:** 2012-11-01

**Authors:** Alexander M. Cardoso, Janaína J. V. Cavalcante, Maurício E. Cantão, Claudia E. Thompson, Roberto B. Flatschart, Arnaldo Glogauer, Sandra M. N. Scapin, Youssef B. Sade, Paulo J. M. S. I. Beltrão, Alexandra L. Gerber, Orlando B. Martins, Eloi S. Garcia, Wanderley de Souza, Ana Tereza R. Vasconcelos

**Affiliations:** 1 Instituto Nacional de Metrologia, Qualidade e Tecnologia, Duque de Caxias, Rio de Janeiro, Brazil; 2 Laboratório Nacional de Computação Científica, Petrópolis, Rio de Janeiro, Brazil; 3 Empresa Brasileira de Pesquisa Agropecuária - CNPSA, Concórdia, Santa Catarina, Brazil; 4 Instituto de Bioquímica Médica, Universidade Federal do Rio de Janeiro, Rio de Janeiro, Rio de Janeiro, Brazil; 5 Instituto de Biofísica Carlos Chagas Filho, Universidade Federal do Rio de Janeiro, Rio de Janeiro, Rio de Janeiro, Brazil; 6 Instituto Oswaldo Cruz, Fundação Oswaldo Cruz, Rio de Janeiro, Rio de Janeiro, Brazil; Swedish University of Agricultural Sciences, Sweden

## Abstract

The shortage of petroleum reserves and the increase in CO_2_ emissions have raised global concerns and highlighted the importance of adopting sustainable energy sources. Second-generation ethanol made from lignocellulosic materials is considered to be one of the most promising fuels for vehicles. The giant snail *Achatina fulica* is an agricultural pest whose biotechnological potential has been largely untested. Here, the composition of the microbial population within the crop of this invasive land snail, as well as key genes involved in various biochemical pathways, have been explored for the first time. In a high-throughput approach, 318 Mbp of 454-Titanium shotgun metagenomic sequencing data were obtained. The predominant bacterial phylum found was *Proteobacteria*, followed by *Bacteroidetes* and *Firmicutes*. *Viruses*, *Fungi*, and *Archaea* were present to lesser extents. The functional analysis reveals a variety of microbial genes that could assist the host in the degradation of recalcitrant lignocellulose, detoxification of xenobiotics, and synthesis of essential amino acids and vitamins, contributing to the adaptability and wide-ranging diet of this snail. More than 2,700 genes encoding glycoside hydrolase (GH) domains and carbohydrate-binding modules were detected. When we compared GH profiles, we found an abundance of sequences coding for oligosaccharide-degrading enzymes (36%), very similar to those from wallabies and giant pandas, as well as many novel cellulase and hemicellulase coding sequences, which points to this model as a remarkable potential source of enzymes for the biofuel industry. Furthermore, this work is a major step toward the understanding of the unique genetic profile of the land snail holobiont.

## Introduction

The use of renewable energy sources in the global energy matrix is important for two main reasons. Firstly, our fossil fuel reserves are diminishing, and the lack of readily available substitutes may result in an energy shortage in the not-so-distant future. Secondly, burning of fossil fuels release large amounts of greenhouse gases (carbon dioxide, nitrogen oxides, sulfur hexafluoride, hydrofluorocarbons and perfluorocarbons) aggravating global warming [1]–[2].

Brazil is a world leader in renewable energy. Here, fuel ethanol has been produced from sugarcane since the 1970s. This was an important step toward the use of sustainable fuels for vehicles with Otto-cycle engines. By law, anhydrous ethanol has also been added to the gasoline sold in gas stations at a level of at least 20% (E20). Other countries have adopted a similar strategy [1], [3]. Despite these mandates, the competitiveness of ethanol versus gasoline on the global market is still low, and more efficient production will be important to ensure its widespread acceptance.

In traditional ethanol production, sugarcane processing results in solid waste called bagasse, which is composed mainly of lignocellulose. Although it can be burned in industrial plants for heating purposes, a large fraction remains unused. Bagasse has a large energy potential, up to 19 MJ/kg [4], but most organisms cannot readily ferment cellulose and lignin. Improved methods to degrade bagasse and other agriculture residues into fermentable sugars would reduce waste and supply the refinery with more sources to produce ethanol without increasing the cultivation area and accompanying costs. Some organisms produce enzymes that can efficiently degrade cellulose. Their identification, improvement, and production for industrial application may contribute to making second-generation ethanol a competitive commodity [5]–[6]. The relevance of the search for new lignocellulolytic enzymes and microorganisms is demonstrated by the increasing number of projects focusing on the symbiotic and mutualistic microbiota from the digestive tracts of animals such as termite (*Nasutitermes* sp.), cow (*Bos taurus*), wallaby (*Macropus eugenii*), and giant panda (*Ailuropoda melanoleuca*) [7]–[11].

Throughout evolution, the digestive systems of animals adapted mechanical, physiological, and chemical methods to improve use of the available diet. In herbivores, enzymes play a vital role in the degradation of raw vegetable fiber, converting it to small units. The resulting oligomers and monomers become available for absorption by the gut epithelium or support the growth of resident microbes, which also produce enzymes that take part in the digestive process or contribute to the supply of nutritional and defense factors essential to the host organism’s health [12].

The giant land snail *Achatina fulica* is a pulmonate mollusk native to sub-Saharan Africa. Due to human interference, prolificacy, physiological adaptability, and ability to eat a broad range of plant species (Table S1), *Achatina* has reached a near-global distribution and can be found close to cities, roads and farms. Because of its voracious appetite and the speed with which it spreads, it is now considered to be the most destructive terrestrial gastropod worldwide [13]–[14]. Despite that, there are surprisingly few studies focusing on this easy-to-handle snail.

The first studies on animal-produced cellulolytic enzymes were performed on snails (*Helix sp.*) at the end of the 19th century [15]. In addition to their own enzymatic repertoire, land snails retain a microbiota that specializes in the rapid hydrolysis of lignocellulosic plant biomass, which contributes to their extraordinary digestive efficiency (up to 80%) [16]. However, the composition and ecological role of the microbiota of land snails, including *A. fulica*, remain largely unexplored.

Unlike other strategies for novel enzyme and microorganism identification, metagenomic analysis offers some clear benefits as a powerful alternative to culture-dependent methods. With the increase in throughput and the development of fast searching algorithms, metagenomic analysis is becoming easier and more efficient. The resulting data may also be continuously submitted to further prospection and evaluation. Thus, metagenomics improves the access to the full genetic potential of a given environment [17].

Here, for the first time we investigated the microbial enzymes within the crop of the giant snail, focusing on those enzymes that are potentially relevant to the degradation of plant biomass into fermentable sugars, using high-throughput sequencing and bioinformatic approaches. Comparison of the carbohydrate active enzymes (CAZy) in the snail crop to a diversity of other biomes including arthropoda, herbivores, omnivores, soil, and aquatic metagenomes using hierarchical clustering revealed the snail’s similarity to the insect pest beetle *Dendroctonus ponderosae*, which indicates similar metabolic capabilities. Furthermore, this work provides insights into the land snail microbiome.

## Materials and Methods

### Sample Collection

Seven wild adult *Achatina fulica* snails were collected by hand in the early morning during the rainy season in March 2008. The snails were collected from a habitat with low evidence of impact from human activities within the city of Rio de Janeiro (22°49′18.83′ ´S; 43°31′30.01′ ´W) in a place covered by humid tropical evergreen broadleaf forest vegetation, the so-called Mata Atlântica [18]. The snails were washed with sterile distilled water and then transported to the laboratory. The crop fluid was collected by cannulation of the mouth-esophagus with a #24 needle-less scalp vein set attached to a syringe by a Luer lock connector [19].

### DNA Extraction

For community metagenome sequencing, total DNA was isolated according to a protocol specific for high molecular weight DNA extraction from environmental samples [20]. Briefly, the pooled crop fluid (15 ml) was filtered first through a 3.0-µm filter and then through a 0.2-µm Sterivex (Millipore) filter to separate free-living microbes from larger organisms, viruses and particles. Total nucleic acid was isolated from the Sterivex by cell lysis with Proteinase K and SDS followed by phenol-chloroform extraction. In addition, the non-filtered crop fluid microbiotic DNA was extracted using the Power Soil DNA Extraction Kit (MoBio), according to the manufacturer’s recommended protocol. DNA concentration, purity, and the overall integrity were checked on a Nanodrop (Thermo Scientific) and by agarose gel electrophoresis.

### Pyrosequencing and Sequence Processing Workflow

Two libraries were prepared by following the instructions from the Rapid Library Preparation Method Manual - GS FLX Titanium Series (454-Roche): one from 500 ng of DNA extracted from the snails’ filtered digestive juice (crop fluid) and another from 500 ng of DNA extracted from non-filtered juice. Titration, emulsion PCR, and sequencing steps were performed according to the manufacturer’s protocol. A two-region 454 sequencing run was performed on a 70×75 PicoTiterPlate (PTP) using the Genome Sequencer FLX System (Roche). Each region was loaded with one of the library preparations. Before sequence analysis and assembly, artificially replicated sequences that are generated as an inherent artifact of 454-based pyrosequencing were identified and removed using the Replicates software [21]. The remaining reads were further filtered to remove short sequences (fewer than 180 bp) or sequences with Phred quality ≤20 using the LUCY program [22].

### Taxonomic Distribution and Functional Analysis

MEGAN4 [23] was used to analyze the taxonomic distribution of each read hit of the BLASTX algorithm [24] against the NCBI-NR protein database. Meta-RNA software [25] was employed to perform 16S rRNA identification and the sequences were classified with RDP Classifier software based on the RDP Database [26]. We analyzed the functional composition of the assembled metagenome data by comparison with the SEED [27] and COG databases [28] available on the MG-RAST server [29] using an e-value ≤1e−5 as a cutoff. Functional comparison against public metagenomes was also performed. To identify statistically significant and biologically meaningful differences between the snail crop and other microbiomes, we employed the two-way Fisher’s exact test with a Benjamin-Hochberg FDR multiple test correction within the Statistical Analysis of Metagenomic Profiles, STAMP [30]. Principal coordinates analysis (PCoA) and Rarefaction curve of annotated species richness were calculated in MG-RAST [28].

Further functional analysis was executed using the KEGG database [31] using MEGAN4 software [23]. The functional annotation and analysis of all contig sequences were also performed using the System for Automated Bacterial Integrated Annotation, SABIA [32]. An automatic annotation was performed using the following criteria: ORFs with BlastP hits on databases KEGG, NCBI-nr, or UniProtKB/Swiss-Prot, subject and query coverage ≥60%, and positives ≥60% were assigned “valid”. ORFs with no BlastP hits found on databases NCBI-nr, KEGG, UniProtKB/Swiss-Prot, TCDB, and Interpro or not included in the criteria above were assigned as “hypothetical”.

Carbohydrate-active enzyme domains assigned by the CAZy database [33] were searched in predicted protein sequences using HMMER 3.0 software [34] against the Pfam 26.0 database [35] using a cutoff of 1e−4. To compare the snail crop CAZyome to other sequenced metagenomes, a comparative analysis was performed using the carbohydrate enzyme content as a comparative metric [36]–[37].

### Comparative Metric Analysis

Host-associated and environmental metagenomes were downloaded from the IMG/M (Integrated Microbial Genomes with Microbiome Samples) database [38], including metagenomes from plant, arthropod, mammal, water, and soil samples. The number of genes in Pfam (obtained through the Pfam count statistical data downloaded for each metagenome) was calculated using a self-written bash script using a BLASTP e-value of 1e−04. The correspondence between Pfam and CAZy was automatically reached using another self-written bash script, resulting in a matrix with the number of reads found in different CAZy families in each metagenome. A new matrix, with each row corresponding to a metagenome and each column corresponding to a CAZy ID, was obtained. The proportion of each CAZy with respect to the total number of annotated CAZy families in that metagenome was calculated, and the resultant value was directed to the appropriate cell of the matrix. Trends in the CAZy family frequencies were examined in SPSS using Principal Component Analysis (PCA) as well as hierarchical clustering [39].

The main idea of the PCA method is to form a new variable (or variables) from a data set, which should contain as much of the variability of that data set as possible, reducing it to a smaller set of uncorrelated variables that will be used in further analysis. Descriptive statistics, such as the mean and standard deviation, were calculated for all variables (CAZy families). The analysis was performed on standardized data based on a correlation matrix. Consequently, the importance of a variable was not determined by the relative size of its variance. The criteria for the number of components to extract included an eigenvalue greater than 1, which indicates that a principal component explains more of the variance than would be expected by chance. The communalities (amount of variance in a variable accounted by all the components) were computed as well as the share of variance of original data explained by each and all components. Hierarchical clustering using Euclidean distances with Ward linkage was performed on standardized data generated from both the proportion matrix and the principal component values obtained by the PCA. The hierarchical clustering analysis allowed us to identify relatively homogeneous clusters of cases based on measured features. The results are displayed in a dendrogram, a hierarchical tree diagram, which shows the linkage points at increasing levels of dissimilarity.

## Results and Discussion

### Snail Anatomy

**Figure 1:**
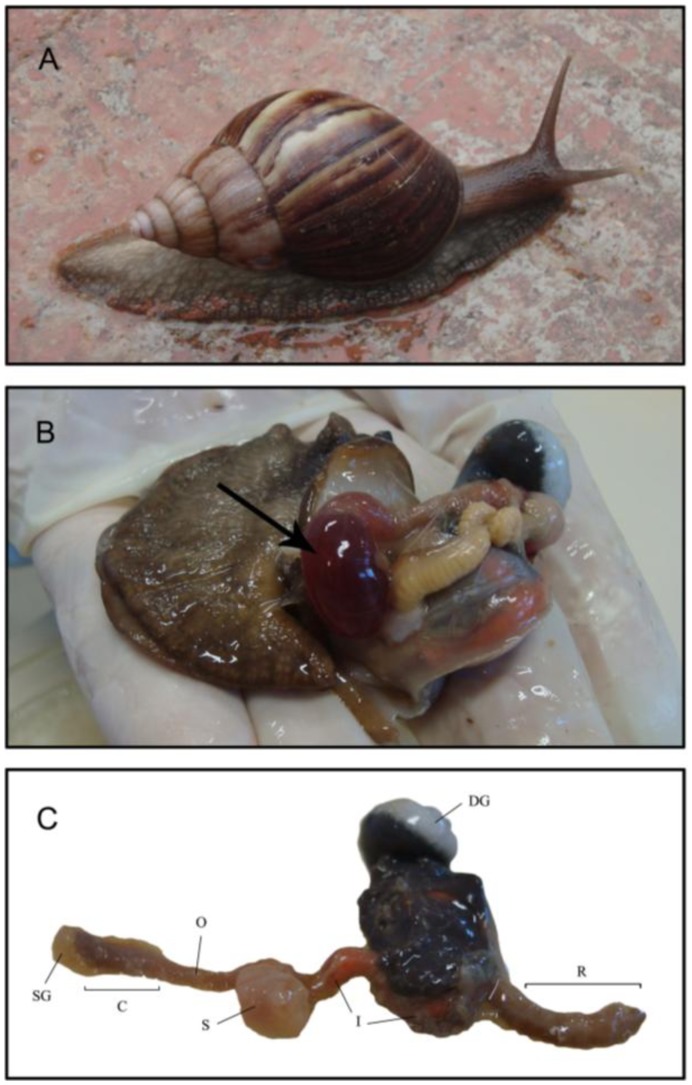
Digestive system of the snail. (A) Photograph of the African giant snail *Achatina fulica*. (B) Whole digestive system dissected. The black arrow indicates the crop filled with juice. DNA for the metagenome analysis was extracted from this fluid. (C) Spread out digestive system of the snail showing the salivary gland (SG), empty crop (C), esophagus (O), stomach (S), intestine (I), digestive gland (DG) and rectum (R).

In *A. fulica* (as in many pulmonate gastropods) the food, scraped by the radula and ingested by the buccal mass, is mixed with the secretions of the salivary gland and accumulates in the crop (ingluvius), a distensible muscular compartment (Figure 1). The crop and stomach is filled via two cannaliculi with the juice produced by the digestive gland (also called hepatopancreas or midgut gland). A muscular valve restricts the passage of the crop contents to the stomach [40]. The medial part of the gut is surrounded by the digestive gland, which secretes more enzymes into the midgut lumen and also absorbs nutrients. The epithelium of the digestive tube is ciliated along almost its entire length, allowing the food to mix with digestive juices and helping transport the alimentary mass. The ciliated epithelia also allow the microbial flora to anchor on the digestive tube [41]. The unabsorbed part of the alimentary mass (bolus) is compacted and passed directly into the rectum.

The snail’s digestion is primarily extracellular [40]. Mapping reducing sugar-producing activity against carboxymethyl cellulose (CMC), avicel, and micronized bagasse [42] along each morpho-functional section of the digestive tube revealed that the crop and the digestive gland have the highest net and specific enzyme activity. To assess the taxonomic, genetic, and functional potential of the microorganisms within *A. fulica*’s crop, a massive random shotgun sequencing effort was performed.

### Overview of the *A. fulica* Metagenome

A total of 1,297,598 reads were produced after raw data processing on 454**-**Titanium pyrosequencer. The Replicates package identified and removed 188,709 reads as replicas. From the remaining 1,108,889 reads, LUCY removed 198,922 low-quality reads, allowing the use of 909,967 sequences for further taxonomic and functional categorization (Table S2). From these sequences, 1,671 were identified as 16S rDNA using the MG-RAST server against RDP, which corresponds to 0.18% of the total sequence obtained. Despite the advantages over 16S libraries, which can show biases in amplification (PCR), reads of 16S rRNA obtained from random DNA shotgun sequencing are not indicated for binning [43]. Other research has reported low assignment values when working with analogous methodologies: 0.06% for the Amazon River metagenome [44] and an average of 0.15% for the bovine rumen metagenome [8].

In addition, another group distribution was performed by MEGAN4, which is software based on the NCBI taxonomy database [23]. Approximately 82% (743,954) of the reads were assigned to a taxon. LCA analysis was considered more flexible for taxonomic classification, identifying more sequences related to *Bacteria*, *Archaea*, *Eukarya,* and *Viruses*. Both approaches generated similar results for bacteria.

In an attempt to decrease redundancies and obtain more complete genes, a restrict assembly was executed using Newbler assembler software version 2.5.3 with the “-rip” flag that allows the user to output each read in only one contig. Reads identified as “problematic” by the GS De Novo Assembler (Partial, Repeat, Outlier, or TooShort) or showing High-Quality Discrepancies (HQD) were filtered out. A new round of assembly and filtering was performed. These steps were repeated until the problematic and high-quality discrepant reads reached the cutoff point of 1% of the total number of reads filtered at the first assembly step. As a result, 423,310 reads remained as singletons while 2,623 contigs ≥500 bp were formed and the average contig size was 4,474 bp. Metagenome data reported in this paper have been deposited into the GenBank database (SRA051264) and on the MG-RAST server (4460151, 4481175, 4480501, and 4482672).

### Analysis of Snail Crop-associated Microbiota

**Figure 2:**
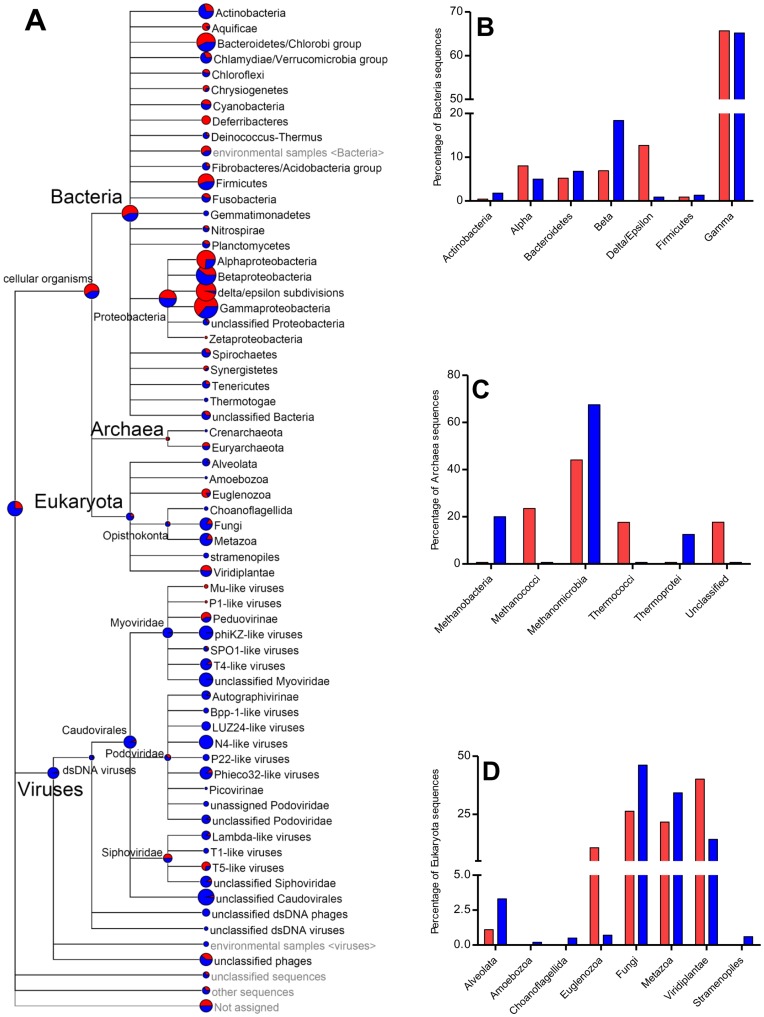
Analysis of composition of the snail microbial community. (A) Phylogenetic diversity of metagenomic sequences, computed by MEGAN4 based on a BLASTX using an e-value cut-off of 1e−5 comparison for filtered (red) and non-filtered (blue) sequences. The size of the circles is scaled logarithmically to represent the number of reads assigned to each taxon. (B) Bacteria, (C) Archaea, and (D) Eukaryota.

Analysis of community composition in filtered crop fluid confirmed an enrichment for prokaryotic populations, thus bypassing the difficulty of interpreting large amounts of repetitive and non-coding eukaryotic sequence data [45]. As expected, the non-filtered crop fluid sample contained more sequences related to *Eukaryota* and *Viruses* (Figure 2; Table S2). No host sequences were observed. The majority of eukaryotic sequences were from *Fungi* (1,618), *Metazoa* (1,218), and *Viridiplantae* (806), which may represent plant DNA contamination (less than 0.088%). A small number of sequences related to the *Alveolata* (108) were identified. Although the role that fungi and protozoa play in cellulose digestion in rumen and lower termites is widely recognized [46]–[47], their exact function in snails remains unknown. Recently, it was shown that the host phylogeny is an important driver that shapes fungal community diversity and composition [48].

The majority of the reads that did match known viruses in the database were from phages that infect bacteria, mostly members of the order *Caudovirales* (Figure 2). Viruses regulate the composition of microbial communities in many environments [49]; however, little is known about the ecological roles that they play in gastrointestinal systems. In the human gut, the viral population seems to be stable and persistant in individuals over time, suggesting that bacteria and viruses are in a mutually beneficial relationship [50].

The distribution of phylotypes and Environmental Gene Tags (EGTs) detected within the crop fluid sample primarily originated from the *Proteobacteria, Bacteroidetes*, and *Firmicutes* groups, consistent with previous SSU rDNA libraries of the snail crop [19]. At the genus level, *Pseudomonas* (37.5%), *Sulfurospirillum* (8.5%), and *Stenotrophomonas* (7.3%) were assigned as principal bacterial groups in the snail crop. Species from these genera are widely distributed in nature, including moist environments, water, soil, and on plants, including fruits and vegetables [51]. Therefore, they can use a varied range of nutrients and may assist the host in the degradation of lignocellulose material, detoxification of organic compounds, and synthesis of amino acids and vitamins.

Previous culture-dependent methodologies have identified cellulolytic *Pseudomonas* and *Stenotrophomonas* species in various invertebrates [52]. Moreover, previous culture-independent studies of bacterial species in the snail crop fluid, based on the 16S rRNA gene sequences, have demonstrated the presence of bacteria related to taxa found in the gut of herbivores, including *Pseudomonas*, *Clostridiaceae, Lactococcus, Bacteroides, Flavobacteriaceae, Mucilaginibacter, Citrobacter, Klebsiella, Aeromonas, Acinetobacter,* and sulfate-reducing bacteria related to *Sulforospirillum* [19]. In both the deepwater sea snail *Alviniconcha hessleri* [53] and the gutless marine oligochaete worm *Olavius algarvensis*, *Epsilonproteobacteria* occur as endosymbionts that serve as an energy source for the host and may participate in the removal of the end products of fermentation [54]; however, such a symbiosis has not yet been described for land snails.

Searches on rRNA databanks (RDP, SILVA, and Greengenes) resulted in no archaeal sequence detection, although BlastX analysis has revealed that genes related to *Euryarchaeota* are present within the snail crop, highlighting how little of this habitat has been described in the literature. Methanogenic archaea are known to be permanent residents in the digestive systems of ruminants, termites, and humans [55]. Although sulfate reduction and homoacetogenesis serve as alternate pathways for removing hydrogen, production of methane may contribute as hydrogen scavengers from the human distal gut [56]–[57].

In addition, sequences associated with *Crenarchaeota*, including the class *Thermoprotei*, have been detected (Figure 2). To the best of our knowledge, this is the first report of the detection of *Archaea*, *Fungi*, and *Viruses* within the crop of a snail. Compared with other metagenomes, the snail microbiota has high species richness (Figure S1), similar to the wallaby foregut and P3 luminal fluid from termite microbiomes. Principal coordinates analysis (PCoA) separated the snail microbiome across axes (Figure S2), indicating diverse and divergent community assemblages inhabiting the crop. In the scatter plot, the first two principal coordinates PC1 and PC2 explained 38.55% and 26.21% of data variation, respectively. PC1 separated snail crop-associated microbial communities from the panda, termite, and chicken microbiomes. PC2 separated from the wallaby and cow rumen environments. This finding suggests that *A. fulica* may represent an unexplored resource of uncharacterized microorganisms and genes.

### Functional Metagenome Analysis

Snail metagenome assembly retrieved 363,365 predicted protein features. Of this total, 294,404 (81.0%) have been assigned an annotation using at least one protein database and 277,079 (94.1% of the annotated features) could be assigned to functional categories. The functional classification based on the COG database was obtained, and the abundance of the main pathways found on the *A. fulica* crop microbiome was compared with those of other metagenomes (Figures S3, S4).

**Figure 3:**
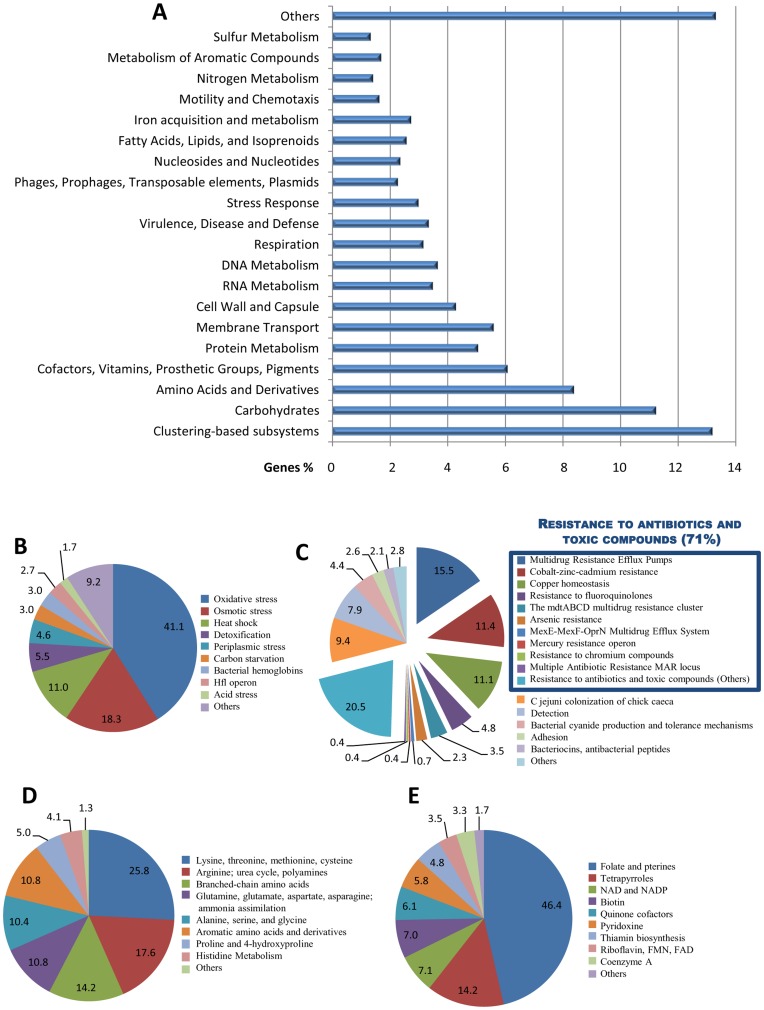
Subsystems-based annotation (SEED) analysis of the snail crop microbiome. A) Percent of genes assigned by MG-RAST to each SEED subsystem. B) Percent of genes in the “Stress Response” subsystem, which includes sequences mainly associated with the protection against oxidative and osmotic stress, as well as heat shock. C) Genes assigned to the “Virulence, Disease and Defense” subsystem. Seventy-one percent of the sequences correspond to genes providing resistance to antibiotics and other toxic compounds, including multi-drug efflux pumps and genes for detoxification of a wide variety of heavy metals (cobalt, zinc, cadmium, chromium, copper and mercury). D) and E) Percent of genes in the subsystems “Amino Acids and Derivatives” and “Cofactors, Vitamins, Prosthetic Groups, Pigments”, respectively.

Subsystems-based annotations (SEED) were performed to gain insights into the metabolic and physiological potential of the snail crop metagenome (Figure 3). In addition to the abundance of genes related to the main metabolic routes: carbohydrate, protein, and nucleic acid (Figure 3A; Figure S3), we found sequences associated with other important processes, such as iron acquisition, nitrogen, phosphorous, sulfur, potassium, cofactor and vitamins pathways, stress response and defense which are more abundant in the snail metagenome, as compared to other gut metagenomes (Figure S4).

The genetic repertoire of the *A. fulica* microbiome includes many sequences associated with protection against oxidative and osmotic stress, heat shock, and metabolism of aromatic compounds (Figure 3B; Figure S5), which could play an important role in the detoxification of phenolic and toxic compounds. Furthermore, we found a great variety of genes coding for antibiotic resistance as well as complete biosynthetic pathways for amino acids and vitamins (Figure 3).

Previous studies based on culture-independent high-throughput technologies have suggested that animals constitute complex biological systems in which mutualistic and symbiotic microbiota can influence host metabolism and complement important physiological functions [58]–[60]. In fact, microbiota that inhabit the gastrointestinal tract of animals considerably affect the growth and health of their hosts by influencing nutrition, pathogenesis, and immunity [61]–[62]. In this context, microbial communities within the snail crop could play an important role in detoxifying phenolic compounds and xenobiotics as well as in the synthesis of essential nutrients and defensive molecules. This relationship may be related to the high physiological adaptability and invasive properties of *A. fulica*.

We also analyzed the functional composition of the snail metagenome using similarity to a non-redundant protein database against KEGG metabolic pathways. This functional assignment revealed the main pathways represented on the microbiome (Figure S6A). A detailed examination of the carbohydrate metabolic pathways (Figure S6B) revealed a significant number (∼7.5%) of putative genes belonging to the subgroup “starch and sucrose metabolism”, including sequences coding for critical enzymes that cover all steps of cellulose deconstruction (Figure S6C).

### Repertoire of Glycoside Hydrolase Functional Domains and Related Modules

To identify how the microbial community associated with *A. fulica* mediates plant fiber degradation, database searches for glycoside hydrolase (GH) domains were performed by HMMER software against the Pfam database. This analysis identified more than 4,000 EGTs from 93 different CAZy (Carbohydrate-Active EnZymes) families [33] and modules belonging predominantly to *Proteobacteria* (Table S3; Figure S7). We found a wide diversity of GH catalytic modules: 2,534 EGTs belonging to 53 different GH families, which indicates an abundance of potential polysaccharide and plant cell wall biomass-degrading enzymes.

We also detected a total of 458 EGTs from 11 families of non-catalytic carbohydrate-binding modules (CBMs), which may promote the interaction of an enzyme with its target substrate, thereby increasing the efficiency of catalysis. This abundance reflects a high capacity to metabolize and degrade diverse lignocellulosic substrates. For example, modules that are likely to bind cellulose were found (CBM4-9-16-22 (PF02018)) [63], but the most frequently occurring module was CBM50 (152 members, with 18% of the sequences belonging to the *Pseudomonas* genus), which is also known as a LysM domain (PF01476). These are modules found attached to enzymes cleaving either chitin or peptidoglycan [64]. This domain is found in many of the enzymes involved in cell wall degradation and is also present in other proteins that are associated with bacterial cell walls. LysM-containing proteins have multiple functions, including modulating host–microbe interactions, such as signaling and recognition, between *Eukarya* and bacterial symbionts [64].

The most represented GH families were GH1 (294 members, with 73% of the sequences belonging to *Gammaproteobacteria*) and GH3 (219 members), which contains a widespread group of glycosidases that cleave non-reducing carbohydrates in oligosaccharides and the side chains of hemicelluloses and pectins. In addition, we found an abundance of GH13 (251 members), which is the most abundant family in metagenomes, such as the termite gut [7], [65], and is the major GH family. This family includes enzymes that have a wide range of substrate specificities and catalytic activities, such as α-amylase, cyclodextrin glycosyltransferase (CGTase), branching enzymes, and cyclomaltodextrinase. The snail microbiome also contained a range of CAZy known to be involved in plant polymer degradation, including cellulases (GH5, GH9), β-galactosidases (GH2, GH27, GH35), chitinases (GH18, GH19), xylanases (GH26, GH43), and CE4 acetylxylan esterases that are required for the complete hydrolysis of xylan, α-xylosidases (GH31), α-mannosidases (GH38), and others targeting pectic polysaccharides, including GH43 and GH62 arabinofuranosidases, GH78 rhamnosidases, CE8 pectin methylesterases, GH28 rhamnogalacturonases, pectate lyases (PL1 and 3), and alginate lyase (PL5). In total, 21 families of glycosyltransferases (782 members), 4 families of carbohydrate esterases (212 members), and 4 families of polysaccharide lyases (94 members) were identified, representing the first and the largest microbial gene catalogue from a terrestrial snail. Moreover, 91 putative full-length genes with a significant match in the KEGG database were identified (Table S3). These include 43 candidate GHs, some of which are possibly involved in cellulose and hemicellulose degradation (such as five putative genes for β-glucosidases from the GH3 family, one putative protein from the GH5 family, and two putative cellulases from the GH8 family).

It is also worth noting the lack of specific CAZy family members, such as genes encoding for the GH7, GH12, GH44, and GH45 endoglucanases, which are essential in many cultured bacteria for cellulose solubilization. Cohesin and dockerin gene modules, which are often found associated with GHs, and are also known as cellulosome-associated modules, were not found in this metagenome. This may be a result of this particular microbiome structure, suggesting that there is still a deficiency in the information about the mechanisms involved in cellulose hydrolysis and dockerin-cohesin mediated complex assemblies in gut microbiomes.

### Trends in the CAZy Family Frequencies

Comparing the snail GH profile with those of herbivores (giant panda, wallaby, and termite) and an omnivore (human), some interesting characteristics and patterns can be observed (Table 1).

**Table 1:** Comparison of the glycoside hydrolase (GH) profiles targeting plant structural polysaccharides in the snail, termite, giant panda, wallaby, and human metagenomes.

CAZy Family	Known activity	SNAIL	TERMITE	WALLABY	PANDA	HUMAN
**Cellulases**						
GH5	cellulase	36	125	27	1	7
GH6	endoglucanase	4	0	0	0	0
GH7	endoglucanase	0	0	0	0	0
GH9	endoglucanase	15	43	5	0	0
GH44	endoglucanase	0	0	0	0	0
GH45	endoglucanase	0	6	0	0	0
GH48	endo-processive cellulases	2	0	0	0	0
Total		57 (2)	174 (16)	32 (4)	1 (0.5)	7 (1)
**Endohemicellulases**						
GH8	endo-xylanases	46	21	2	1	2
GH10	endo-1,4-β-xylanase	25	102	19	1	2
GH11	xylanase	1	19	0	0	0
GH12	endoglucanase & xyloglucan hydrolysis	0	0	0	0	0
GH26	β-mannanase & xylanase	11	20	8	0	1
GH28	galacturonases	69	15	10	0	3
GH53	endo-1,4-β-galactanase	9	20	11	4	11
Total		161 (6)	197 (18)	50 (6)	6 (2.5)	19 (3)
**Cell wall elongation**						
GH16	xyloglucanases & xyloglycosyltransferases	12	6	6	6	1
GH17	1,3-β-glucosidases	2	0	0	0	0
GH81	1,3-β-glucanase	1	0	0	0	0
Total		15 (1)	6 (1)	6 (1)	6 (3)	1 (0)
**Debranching enzymes**						
GH51	α-L-arabinofuranosidase	22	13	19	2	15
GH62	α-L-arabinofuranosidase	2	0	0	0	0
GH67	α-glucuronidase	5	6	1	2	1
GH78	α-L-rhmnosidase	73	7	46	1	13
Total		102 (4)	26 (2)	66 (8)	5 (2)	29 (5)
**Oligosaccharide-degrading enzymes**						
GH1	β-glucosidase & many other β-linked dimers	294	27	94	41	54
GH2	β-galactosidases & other β-linked dimer	66	32	39	4	29
GH3	mainly β-glucosidases	219	109	101	11	55
GH29	α-L-fucosidase	70	12	5	0	7
GH35	β-galactosidase	32	7	8	1	4
GH38	α-mannosidase	18	18	3	8	6
GH39	β-xylosidase	6	13	3	8	2
GH42	β-galactosidase	54	33	17	7	15
GH43	arabinases & xylosidases	185	63	72	13	34
GH52	β-xylosidase	0	3	0	0	0
Total		944 (36)	317 (28)	342 (39)	93 (41)	206 (36)

Data are presented with the GHs grouped according to their major function roles in the degradation of plant fiber, as classified in Allgaier *et al*., 2010 [70]. The numbers in parentheses represent the percentages of these groups relative to the total number of GHs identified in the metagenomic datasets [2590 for snail, 872 for wallaby, 1117 for termite, 227 for panda (from 3 samples), and for human (from 2 samples)].

First, the fraction of annotated carbohydrate active enzymes in the snail crop assigned to the oligosaccharide degradation category from the snail (36%) was very similar to that observed in samples from the giant panda, wallaby, and human. The profile of debranching enzymes from the snail metagenome (4%), while most similar to that of the termite and panda (and lower than that of the wallaby), showed higher relative abundance of GH78 rhamnosidases, which include enzymes with α-L-rhamnosidase activity. These enzymes are very interesting in terms of their suitability for various biotechnological applications within the food, pharmaceutical, and chemical industries and in the detoxification of rhamnose-conjugated plant secondary metabolites [66]. Nevertheless, the fraction of cellulases and endohemicellulases was higher in the snail than the levels observed in the giant panda and human but still lower than that of the termite indicating that despite its preference for fresh plants and fruits, the snail may be able to consume and degrade more recalcitrant materials with the help of its microbiota. These results suggest that the snail holobiont represents a novel and interesting model for studying the degradation of lignocellulose.

**Figure 4:**
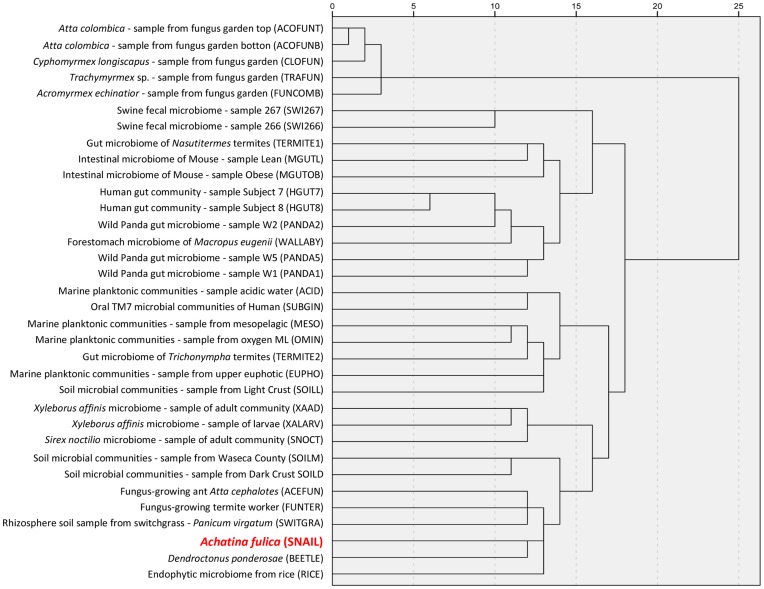
Dendrogram showing the relationships among metagenomes obtained through SPSS hierarchical clustering analysis. *Achatina fulica* crop microbiome (SNAIL) was compared with fungus garden microbial communities from *Atta colombica* in top (ACOFUNT) and bottom (ACOFUNB), *Atta cephalotes* (ACEFUN), *Cyphomyrmex longiscapus* (CLOFUN), *Trachymyrmex* (TRAFUN), and *Acromyrmex echinatior* (FUNCOMB); fecal microbiome of swine (SWI267 and SWI266); P3 luminal contents of *Nasutitermes* (TERMITE1); intestinal micobiome of lean (MGUTL) and obese (MGUTOB) mice; fecal microbiome of healthy adult humans (HGUT7 and HGUT8); feces from wild pandas (PANDA2, PANDA5, and PANDA1); forestomach from *Macropus eugenii* (WALLABY); Richmond acid mine drainage (ACID); oral microbiome from humans (SUBGIN); aquatic environment from mesopelagic (MESO), oxygen minimum layer (OMIN), and planktonic zones (EUPHO) in Hawaii; microbiome of *Trichonympha* from termites (TERMITE2); soil microbial communities from farm silage (SOILM), dark crust (SOILD), distinct crusts (SOILL), and switchgrass rhizosphere (SWITGRA); *Xyleborus affinis* from adult (XAAD) and larvae (XALARY); *Sirex noctilio* (SNOCT); fungus-growing termite (FUNTER); *Dendroctonus ponderosae* (BEETLE); and endophytic microbiome from rice (RICE).

The dendrogram obtained by hierarchical clustering shows many clusters from identical or similar biomes (Figure 4). Upon analysis of the host-associated metagenomes, we found that the majority of samples from fungus-growing ants clustered together, including those from *Atta colombica* (ACOFUNT, ACOFUNB), *Cyphomyrmex longiscapus* (CLOFUN), and the *Trachymyrmex* (TRAFUN) genus. The microbiome of these herbivore insects is responsible for the degradation of plant biomass and simulates the role of the gut in other plant biomass degradation systems, such as those found in the termite, cow, panda, and wallaby [37], [67].

A bigger cluster included the human fecal (HGUT7, HGUT8), mouse intestinal (MGUTL, MGUTOB), swine fecal (SWIN266, SWIN267), wild panda gut (PANDA1, PANDA2, PANDA5), and wallaby forestomach (WALLABY) microbiomes. In the same cluster, a termite gut microbiome (TERMITE1) was found. The wallaby metagenome was closer to the panda than to the human or swine metagenomes. The macropods are herbivores and possess unique adaptations to this diet, such as a forestomach where host-microbe associations allows the digestion of hemicellulose and cellulose [10]. Despite the fact that giant pandas do not have the long intestinal tract typical of other herbivores, it has been demonstrated that their gut microbiome contains putative cellulose-metabolizing symbionts [11]. Consequently, the similarity between the wallaby and panda microbiomes is not surprising. Humans, mice, and swine share similar genetic complexity and omnivorous feeding behavior [7], [68], which explains why their metagenomes were placed together in the major cluster. However, previous work has demonstrated that the cell wall and capsule profiles in the swine samples are more similar to those from the termite than those from the human gut [68]. This result was confirmed by our metagenomic comparison using carbohydrate-active enzymes, indicating similar metabolic capabilities between these microbiomes.

Another group was formed from four aquatic metagenomes (MESO, EUPHO, OMIN, ACID) as well as soil (SOILL), human oral (SUBGIN), and a protist microbiome that lives in the intestine of the termite (TERMITE2). The oral microbial communities were close to the acidic water, showing the similarities among communities belonging to acidic environments. A smaller cluster was formed by *Sirex noctilio* (SNOCT), adult (XAAD) and larvae (XALARV) *Xyleborus affinis* microbiomes, which are arthropod host-associated.

Finally, a cluster including microbiota associated with Arthropoda (ACEFUN, FUNTER, BEETLE), two plant-host associated (RICE, SWITCHGRA), and the snail metagenome were identified and found to be similar. Two soil samples (SOILD, SOILM) formed an external group. Clustering analysis of CAZy profiles showed that the microbiota from the crop of the giant snail is closest to microbial communities from the beetle *Dendroctonus ponderosae.* Native to the forests of western North America, this beetle is the most important insect pest of pine and is responsible for the loss of millions of trees. These beetles attack trees by boring tunnels under the bark. Consequently, their microbiota may reveal important aspects of the cellulosic plant biomass conversion. Additionally, the similarity to the endophytic microbiome from rice and to microbial communities from the switchgrass (*Panicum virgatum*) rhizosphere may be due to the snail’s feeding habits. In these environments, hydrolytic plant polymer-degrading enzymes have been demonstrated to be an important characteristic for the establishment of endophytes in their hosts, resulting in a high abundance of cellulases, xylanases, hydrolases, cellulose-binding proteins, and pectinases [69].

## Concluding Remarks

Deconstructing plant cell walls to improve the efficiency of processing biomass from bagasse and other agriculture residues by enzymatic hydrolysis is considered the most promising environmentally friendly technology available for second-generation ethanol production. However, the heterogeneity and complexity of cell wall structural components and the presence of inhibitors of these enzymes make industrial scale-up a difficult task. Presented in this study is a comparative metagenome analysis of the first land snail crop microbiome from a highly invasive species, which has a wide-ranging diet and is capable of consuming different varieties of plants and substrates. The *A. fulica* holobiont represents a prosperous reservoir of novel GH genes and related modules, some of which have not been previously detected in other metagenomic datasets, representing a remarkable and unique complexity and diversity of genes. Moreover, the diverse metabolic capabilities of the snail metagenome may provide important physiological functions that assist the host in adapting successfully to different environments and feeding habitats. In these circumstances, we anticipate that future explorations of the land snail microbiome will contribute not only to our understanding of basic biology and host-microbiota co-evolution but also to areas of biotechnological relevance, such as discovery and bioengineering of lignocellulolytic enzymes, for use in biomass-to-bioethanol applications to develop a more eco-friendly biofuel.

## Supporting Information

Figure S1 Rarefaction curve of annotated species richness. The curves represent the average number of different species annotations for subsamples of the complete dataset calculated in MGRAST.
(TIF)

Figure S2 Principal coordinates analysis (PCoA) for giant snail (this study), wallaby, giant panda, chicken, termite, and cow rumen metagenomes. The data were compared with RDP using a maximum e-value of 1e−5, a minimum alignment length of 50, normalized values, and bray-curtis distances calculated in MGRAST.
(TIF)

Figure S3 Metabolic comparison according to the clusters of orthologous groups’ functional categories. The color scale corresponds to the relative number of putative genes within each metagenome.
(TIF)

Figure S4 Extended error bar of functional gene groups from *Achatina fulica* versus other gut metagenomes. Pair-wise comparisons were calculated for the snail metagenome versus (A) termite, (B) cow rumen, (C) panda, (D) wallaby, (E) chicken, and (F) human metagenomes are shown.
(TIF)

Figure S5 Functional analysis (SEED) for Metabolism of Aromatic Compounds category.
(TIF)

Figure S6 Functional analysis based on the KEGG database. A) Relative number of genes from the snail crop metagenome classified according to the KEGG main categories. As expected, the sequences related to carbohydrate and amino acid metabolism predominate, accounting for 17% and 15% of total coding sequences related to KEGG pathways, respectively. B) Examination of the relative gene diversity within the KEGG pathway for carbohydrate metabolism. C) KEGG sub-pathway for starch and sucrose metabolism. The color scale represents the number of genes (in logarithmic scale) found for each KEGG entry in the giant snail metagenome. Notice that a great variety of putative genes are related to cellulose degradation, including 49 endo-1,4-β-D-glucanases (entry 3.2.1.4), 3 types of 1,4-β-cellobiosidases (3.2.1.91), and 390 β-glucosidases (3.2.1.21). Furthermore, 27 sequences were classified as putative xylan-degrading enzymes (3.2.1.37, 1,4-β-xylosidases).
(TIF)

Figure S7 Contribution of organism taxa (Class level) to glycoside hydrolase (GH) hits on *Achatina fulica* metagenome. The color represents the proportion of each organism shown relative to the number of each GH hit. The scale is linear; red indicates 0, and blue indicates 100%.
(TIF)

Table S1 Food preferences of *Achatina fulica*.
(XLS)

Table S2 Summary of metagenomic data obtained from the *Achatina fulica* crop microbiome.
(DOC)

Table S3 Inventory of putative glycoside hydrolases (GH), polysaccharide lyases (PL), glycosyltransferases (GT), carbohydrate esterases (CE) and carbohydrate-binding modules (CBM) identified in the snail crop microbiome.
(XLS)

## References

[1] Goldemberg J (2007) Ethanol for a sustainable energy future. Science 315: 808–810. 17289989 10.1126/science.1137013

[2] Somerville C, Youngs H, Taylor C, Davis SC, Long SP (2010) Feedstocks for Lignocellulosic Biofuels. Science 329: 790–792. 20705851 10.1126/science.1189268

[3] Goldemberg J (2008) The Brazilian biofuels industry. Biotechnol Biofuels 1: 6–13. 18471272 10.1186/1754-6834-1-6 PMC2405774

[4] Jenkins BM, Baxter LL, Miles-Jr TR, Miles TR (1998) Combustion properties of biomass. Fuel Proc Technol 54: 17–46.

[5] Ragauskas AJ, Williams CK, Davison BH, Britovsek G, Cairney J, et al (2006) The Path Forward for Biofuels and Biomaterials. Science 311: 484–489. 16439654 10.1126/science.1114736

[6] Rubin EM (2008) Genomics of cellulosic biofuels. Nature 454: 841–845. 18704079 10.1038/nature07190

[7] Warnecke F, Luginbühl P, Ivanova N, Ghassemian M, Richardson TH, et al (2007) Metagenomic and functional analysis of hindgut microbiota of a wood-feeding higher termite. Nature 450: 560–565. 18033299 10.1038/nature06269

[8] Brulc JM, Antonopoulos DA, Miller ME, Wilson MK, Yannarell AC, et al (2009) Gene-centric metagenomics of the fiber-adherent bovine rumen microbiome reveals forage specific glycoside hydrolases. Proc Natl Acad Sci USA 106: 1948–1953. 19181843 10.1073/pnas.0806191105 PMC2633212

[9] Hess M, Sczyrba A, Egan R, Kim TW, Chokhawala H, et al (2011) Metagenomic discovery of biomass-degrading genes and genomes from cow rumen. Science 331: 463–467. 21273488 10.1126/science.1200387

[10] Pope PB, Denman SE, Jones M, Tringe SG, Barry K, et al (2010) Adaptation to herbivory by the Tammar wallaby includes bacterial and glycoside hydrolase profiles different from other herbivores. Proc Natl Acad Sci USA 107: 14793–14798. 20668243 10.1073/pnas.1005297107 PMC2930436

[11] Zhu L, Wu Q, Dai J, Zhang S, Wei F (2011) Evidence of cellulose metabolism by the giant panda gut microbiome. Proc Natl Acad Sci USA 108: 17714–17719. 22006317 10.1073/pnas.1017956108 PMC3203778

[12] Karasov WH, Martínez del Rio C, Caviedes-Vidal E (2011) Ecological physiology of diet and digestive systems. Annu Rev Physiol 73: 69–93. 21314432 10.1146/annurev-physiol-012110-142152

[13] Thiengo SC, Faraco FA, Salgado NC, Cowie RH, Fernandez MA (2007) Rapid spread of an invasive snail in South America: the giant African snail, *Achatina fulica*, in Brasil. Biol Invasions 9: 693–702.

[14] Albuquerque FS, Peso-Aguiar MC, Assunção-Albuquerque MJT (2008) Distribution, feeding behavior and control strategies of the exotic land snail *Achatina fulica* (Gastropoda: Pulmonata) in the northeast of Brazil. Braz J Biol 68: 837–842. 19197503 10.1590/s1519-69842008000400020

[15] Biedermann W, Moritz P (1898) Beiträge zur vergleichenden Physiologie der Verdauung II. Ueber ein celluloselösendes Enzym im Lebersecret der Schnecke (*Helix pomatia*). Pflüg Arch 73: 219–287.

[16] Charrier M, Daguzan J (1980) Food consumption: production and energy budget in *Helix aspersa* Müller (Gastropoda Pulmonata). Ann Nutr Alim 34: 147–166. 7416661

[17] Tringe SG, Rubin EM (2005) Metagenomics: DNA sequencing of environmental samples. Nature Rev Gen 6: 805–814. 10.1038/nrg1709 16304596

[18] Oliveira-Filho AT, Fontes MAL (2000) Patterns of floristic differentiation among Atlantic Forests in Southeastern Brazil and the influence of climate. Biotropica 32: 793–810.

[19] Cardoso AM, Cavalcante JJ, Vieira RP, Lima JL, Grieco MA, et al (2012) Gut bacterial communities in the giant land snail *Achatina fulica* and their modification by sugarcane-based diet. PloS ONE 7: e33440. 22438932 10.1371/journal.pone.0033440 PMC3305317

[20] Vieira RP, Gonzalez AM, Cardoso AM, Oliveira DN, Albano RM, et al (2008) Relationships between bacterial diversity and environmental variables in a tropical marine environment, Rio de Janeiro. Environ Microbiol 10: 189–199. 17892478 10.1111/j.1462-2920.2007.01443.x

[21] Gomez-Alvarez V, Teal TK, Schmidt TM (2009) Systematic artifacts in metagenomes from complex microbial communities. ISME J 3: 1314–1317. 19587772 10.1038/ismej.2009.72

[22] Chou HH, Holmes MH (2001) DNA sequence quality trimming and vector removal. Bioinformatics 17: 1093–1104. 11751217 10.1093/bioinformatics/17.12.1093

[23] Huson DH, Mitra S, Ruscheweyh HJ, Weber N, Schuster SC (2011) Integrative analysis of environmental sequences using MEGAN4. Genome Res 21: 1552–1560. 21690186 10.1101/gr.120618.111 PMC3166839

[24] Altschul SF, Madden TL, Schäffer AA, Zhang J, Zhang Z, et al (1997) Gapped BLAST and PSI-BLAST: a new generation of protein database search programs. Nucleic Acids Res 25: 3389–3402. 9254694 10.1093/nar/25.17.3389 PMC146917

[25] Huang Y, Gilna P, Li W (2009) Identification of ribosomal RNA genes in metagenomic fragments. Bioinformatics 25: 1338–1340. 19346323 10.1093/bioinformatics/btp161 PMC2677747

[26] Cole JR, Wang Q, Cardenas E, Fish J, Chai B, et al (2009) The Ribosomal Database Project: improved alignments and new tools for rRNA analysis. Nucleic Acids Res 37: 141–145. 10.1093/nar/gkn879 PMC2686447 19004872

[27] Overbeek R, Begley T, Butler RM, Choudhuri JV, Chuang HY, et al (2005) The subsystems approach to genome annotation and its use in the project to annotate 1000 genomes. Nucleic Acids Res 33: 5691–5702. 16214803 10.1093/nar/gki866 PMC1251668

[28] Tatusov RL, Koonin EV, Lipman DJ (1997) A genomic perspective on protein families. Science 278: 631–637. 9381173 10.1126/science.278.5338.631

[29] Meyer F, Paarmann D, D’Souza M, Olson R, Glass EM, et al (2008) The metagenomics RAST server - a public resource for the automatic phylogenetic and functional analysis of metagenomes. BMC Bioinformatics 9: 386–394. 18803844 10.1186/1471-2105-9-386 PMC2563014

[30] Parks DH, Beiko RG (2010) Identifying biologically relevant differences between metagenomic communities. Bioinformatics 26: 715–721. 20130030 10.1093/bioinformatics/btq041

[31] Kanehisa M, Goto S, Sato Y, Furumichi M, Tanabe M (2012) KEGG for integration and interpretation of large-scale molecular data sets. Nucleic Acids Res 40: 109–114. 10.1093/nar/gkr988 PMC3245020 22080510

[32] Almeida LG, Paixão R, Souza RC, Costa GC, Barrientos FJ, et al (2004) A System for Automated Bacterial (genome) Integrated Annotation - SABIA. Bioinformatics 20: 2832–2833. 15087310 10.1093/bioinformatics/bth273

[33] Cantarel BL, Coutinho PM, Rancurel C, Bernard T, Lombard V, et al (2009) The Carbohydrate-Active EnZymes database (CAZy): an expert resource for Glycogenomics. Nucleic Acids Res 37: 233–238. 10.1093/nar/gkn663 PMC2686590 18838391

[34] Finn RD, Clements J, Eddy SR (2011) HMMER web server: interactive sequence similarity searching. Nucleic Acids Res 39: 29–37. 10.1093/nar/gkr367 PMC3125773 21593126

[35] Punta M, Coggill PC, Eberhardt RY, Mistry J, Tate J, et al (2012) The Pfam protein families database. Nucleic Acids Res 40: 290–301. 22127870 10.1093/nar/gkr1065 PMC3245129

[36] Tringe SG, von Mering C, Kobayashi A, Salamov AA, Chen K, et al (2005) Comparative metagenomics of microbial communities. Science 308: 554–557. 15845853 10.1126/science.1107851

[37] Suen G, Scott JJ, Aylward FO, Adams SM, Tringe SG, et al (2010) An insect herbivore microbiome with high plant biomass-degrading capacity. PLoS Genet 6: e1001129. 20885794 10.1371/journal.pgen.1001129 PMC2944797

[38] Markowitz VM, Ivanova NN, Szeto E, Palaniappan K, Chu K, et al (2008) IMG/M: a data management and analysis system for metagenomes. Nucleic Acids Res 36: 534–538. 10.1093/nar/gkm869 PMC2238950 17932063

[39] Willner D, Thurber RV, Rohwer F (2009) Metagenomic signatures of 86 microbial and viral metagenomes. Environ Microbiol 11: 1752–1766. 19302541 10.1111/j.1462-2920.2009.01901.x

[40] Ghose KC (1963) The alimentary system of *Achatina fulica* . Trans Am Microsc Soc 82: 149–167.

[41] Zbinden M, Pailleret M, Ravaux J, Gaudron SM, Hoyoux C, et al (2010) Bacterial communities associated with the wood-feeding gastropod *Pectinodonta* sp. (Patellogastropoda, Mollusca). FEMS Microbiol Ecol 74: 450–463. 20831591 10.1111/j.1574-6941.2010.00959.x

[42] Lucena SA, Lima LS, Cordeiro LS Jr, Sant’anna C, Constantino R, et al (2011) High throughput screening of hydrolytic enzymes from termites using a natural substrate derived from sugarcane bagasse. Biotechnol Biofuels 4: 51–60. 22081987 10.1186/1754-6834-4-51 PMC3245446

[43] Saeed I, Tang SL, Halgamuge SK (2012) Unsupervised discovery of microbial population structure within metagenomes using nucleotide base composition. Nucl Acid Res 40: e34. 10.1093/nar/gkr1204 PMC3300000 22180538

[44] Ghai R, Rodriguez-Valera F, McMahon KD, Toyama D, Rinke R, et al (2011) Metagenomics of the water column in the pristine upper course of the Amazon river. PLoS ONE 6: e23785. 21915244 10.1371/journal.pone.0023785 PMC3158796

[45] Kunin V, Copeland A, Lapidus A, Mavromatis K, Hugenholtz P (1998) A Bioinformatician’s Guide to Metagenomics. Microbiol Mol Biol Rev 72: 557–578. 10.1128/MMBR.00009-08 PMC2593568 19052320

[46] Hungate RE (1975) The rumen microbial ecosystem. Ann Rev Ecol Syst 6: 39–66.

[47] Breznak JA, Brune A (1994) Role of Microorganisms in the Digestion of Lignocellulose by Termites. Ann Rev Entomol 39: 453–487.

[48] Liggenstoffer AS, Youssef NH, Couger MB, Elshahed MS (2010) Phylogenetic diversity and community structure of anaerobic gut fungi (phylum *Neocallimastigomycota*) in ruminant and non-ruminant herbivores. ISME J 4: 1225–1235. 20410935 10.1038/ismej.2010.49

[49] Breitbart M, Rohwer F (2005) Here a virus, there a virus, everywhere the same virus? Trends Microbiol 13: 278–284. 15936660 10.1016/j.tim.2005.04.003

[50] Reyes A, Haynes M, Hanson N, Angly FE, Heath AC, et al (2010) Viruses in the faecal microbiota of monozygotic twins and their mothers. Nature 466: 334–338. 20631792 10.1038/nature09199 PMC2919852

[51] Madigan MT, Martinko JM, Parker J (2003). Brock Biology of Microorganisms. 10th ed. Pearson Education, Inc. Upper Saddle River, NJ.

[52] Huang S, Sheng P, Zhang H (2012) Isolation and Identification of Cellulolytic Bacteria from the Gut of *Holotrichia parallela* Larvae (*Coleoptera: Scarabaeidae*) Int J Mol Sci 13, 2563–2577. 10.3390/ijms13032563 PMC3317674 22489111

[53] Suzuki Y, Sasaki T, Suzuki M, Nogi Y, Miwa T, et al (2005) Novel chemoautotrophic endosymbiosis between a member of the *Epsilonproteobacteria* and the hydrothermal-vent gastropod *Alviniconcha* aff. *hessleri* (*Gastropoda: Provannidae*) from the Indian Ocean. Appl Environ Microbiol 71: 5440–5450. 16151136 10.1128/AEM.71.9.5440-5450.2005 PMC1214688

[54] Dubilier N, Mülders C, Ferdelman T, de Beer D, Pernthaler A, et al (2001) Endosymbiotic sulphate-reducing and sulphide-oxidizing bacteria in an oligochaete worm. Nature 411: 298–302. 11357130 10.1038/35077067

[55] Thauer RK, Kaster AK, Seedorf H, Buckel W, Hedderich R (2008) Methanogenic archaea: ecologically relevant differences in energy conservation. Nat Rev Microbiol 6: 579–591. 18587410 10.1038/nrmicro1931

[56] Stams AJ (1994) Metabolic interactions between anaerobic bacteria in methanogenic environments. Antonie Van Leeuwenhoek 66: 271–294. 7747937 10.1007/BF00871644

[57] Gill SR, Pop M, Deboy RT, Eckburg PB, Turnbaugh PJ, et al (2006) Metagenomic analysis of the human distal gut microbiome. Science 312: 1355–1359. 16741115 10.1126/science.1124234 PMC3027896

[58] Dethlefsen L, McFall-Ngai M, Relman DA (2007) An ecological and evolutionary perspective on human-microbe mutualism and disease. Nature 449: 811–818. 17943117 10.1038/nature06245 PMC9464033

[59] Li M, Wang B, Zhang M, Rantalainen M, Wang S, et al (2008) Symbiotic gut microbes modulate human metabolic phenotypes. Proc Natl Acad Sci USA 105: 2117–2122. 18252821 10.1073/pnas.0712038105 PMC2538887

[60] Qin J, Li R, Raes J, Arumugam M, Burgdorf KS, et al (2010) A human gut microbial gene catalogue established by metagenomic sequencing. Nature 464: 59–65. 20203603 10.1038/nature08821 PMC3779803

[61] Turnbaugh PJ, Ley RE, Mahowald MA, Magrini V, Mardis ER, et al (2006) An obesity-associated gut microbiome with increased capacity for energy harvest. Nature. 444: 1027–1031. 10.1038/nature05414 17183312

[62] Qu A, Brulc JM, Wilson MK, Law BF, Theoret JR, et al (2008) Comparative metagenomics reveals host specific metavirulomes and horizontal gene transfer elements in the chicken cecum microbiome. PLoS ONE 3: e2945. 18698407 10.1371/journal.pone.0002945 PMC2492807

[63] Bayer EA, Shoham Y, Lamed R (2006) in Prokaryotes, eds Dworkin M, Falkow S, Rosenberg E, Schleifer K-H, Stackebrandt E (Springer-Verlag, New York), 578–617.

[64] Buist G, Steen A, Kok J, Kuipers OP (2008) LysM, a widely distributed protein motif for binding to (peptido)glycans. Mol Microbiol 68: 838–847. 18430080 10.1111/j.1365-2958.2008.06211.x

[65] Li LL, McCorkle SR, Monchy S, Taghavi S, van der Lelie D (2009) Bioprospecting metagenomes: glycosyl hydrolases for converting biomass. Biotechnol Biofuels 2: 10. 19450243 10.1186/1754-6834-2-10 PMC2694162

[66] Manzanares P, Vallés S, Ramón D, Orejas M (2007) in *Industrial Enzymes*, eds Polaina J, MacCabe AP (Springer Verlag, Berlin-Heidelberg), 117–140.

[67] Scott JJ, Budsberg KJ, Suen G, Wixon DL, Balser TC, et al (2010) Microbial community structure of leaf-cutter ant fungus gardens and refuse dumps. PloS ONE 5: e9922. 20360970 10.1371/journal.pone.0009922 PMC2847949

[68] Lamendella R, Domingo JW, Ghosh S, Martinson J, Oerther DB (2011) Comparative fecal metagenomics unveils unique functional capacity of the swine gut. BMC Microbiol 11: 103. 21575148 10.1186/1471-2180-11-103 PMC3123192

[69] Sessitsch A, Hardoim P, Döring J, Weilharter A, Krause A, et al (2010) Functional characteristics of an endophyte community colonizing rice roots as revealed by metagenomic analysis. Mol Plant Microbe Interact 25: 28–36. 10.1094/MPMI-08-11-0204 21970692

[70] Allgaier M, Reddy A, Park JI, Ivanova N, D’haeseleer P, et al (2010) Targeted Discovery of Glycoside Hydrolases from a Switchgrass-Adapted Compost Community. PLoS ONE 5: e8812. 20098679 10.1371/journal.pone.0008812 PMC2809096

