# Supplementary figures and images for: Metagenomic Analysis of the Microbiota from the Crop of an Invasive Snail Reveals a Rich Reservoir of Novel Genes
Source: PLoS One. 2012 Nov 1;7(11):e48505. doi: 10.1371/journal.pone.0048505 (PMC3486852; doi:10.1371/journal.pone.0048505)

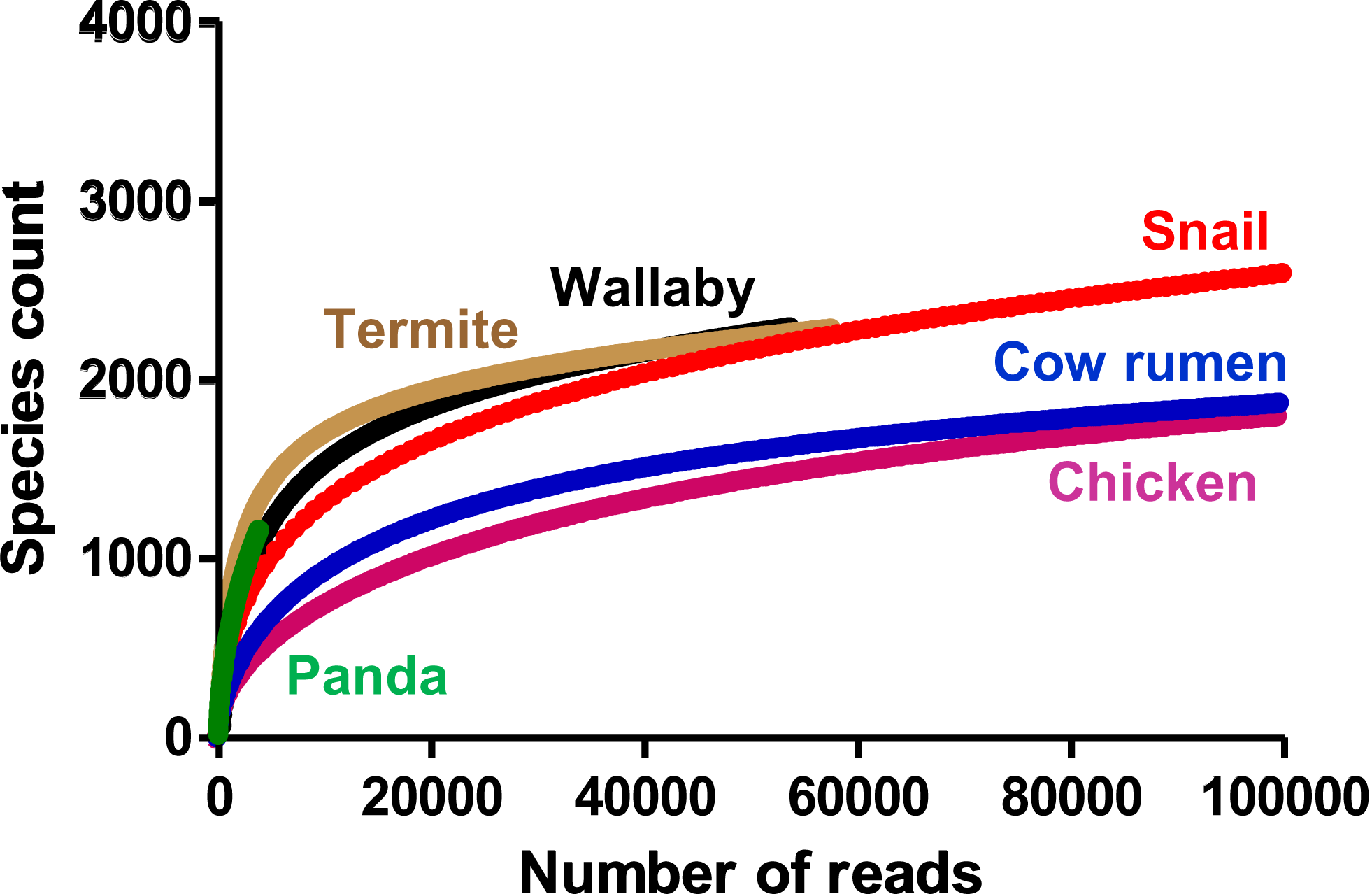

Supplement: Figure S1 — Rarefaction curve of annotated species richness. The curves represent the average number of different species annotations for subsamples of the complete dataset calculated in MGRAST. (TIF) [file pone.0048505.s001.tif]

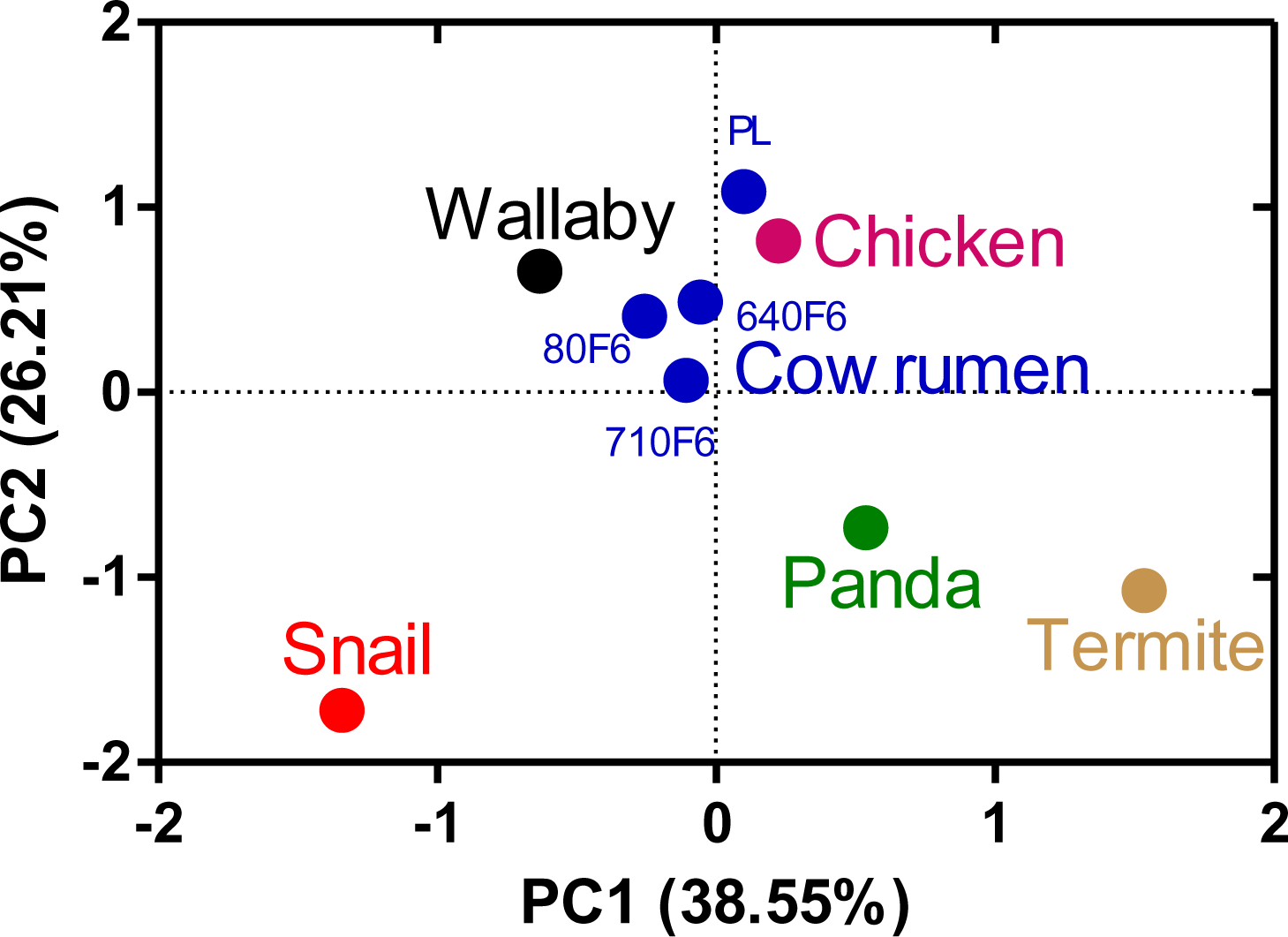

Supplement: Figure S2 — Principal coordinates analysis (PCoA) for giant snail (this study), wallaby, giant panda, chicken, termite, and cow rumen metagenomes. The data were compared with RDP using a maximum e-value of 1e−5, a minimum alignment length of 50, normalized values, and bray-curtis distances calculated in MGRAST. (TIF) [file pone.0048505.s002.tif]

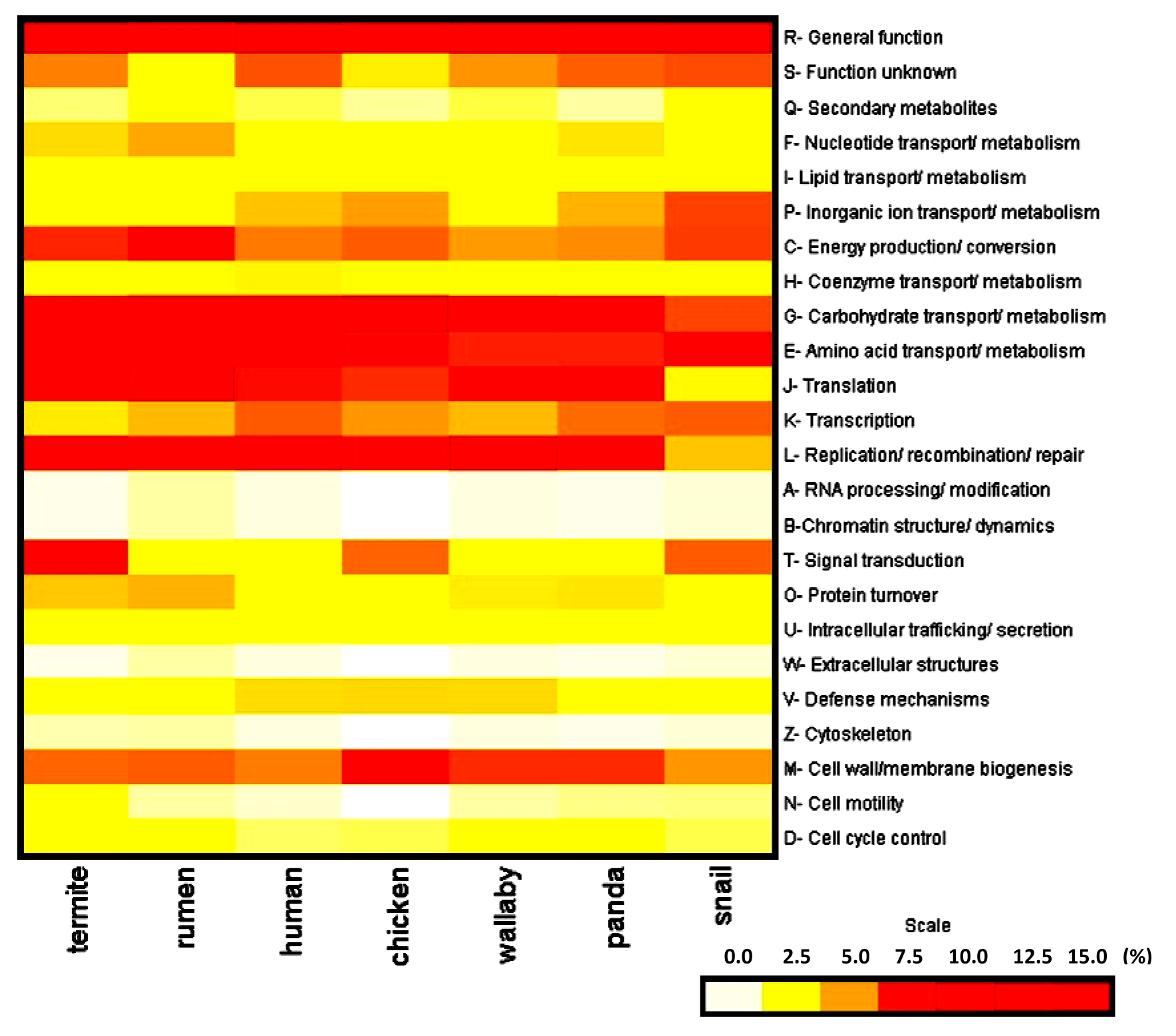

Supplement: Figure S3 — Metabolic comparison according to the clusters of orthologous groups’ functional categories. The color scale corresponds to the relative number of putative genes within each metagenome. (TIF) [file pone.0048505.s003.tif]

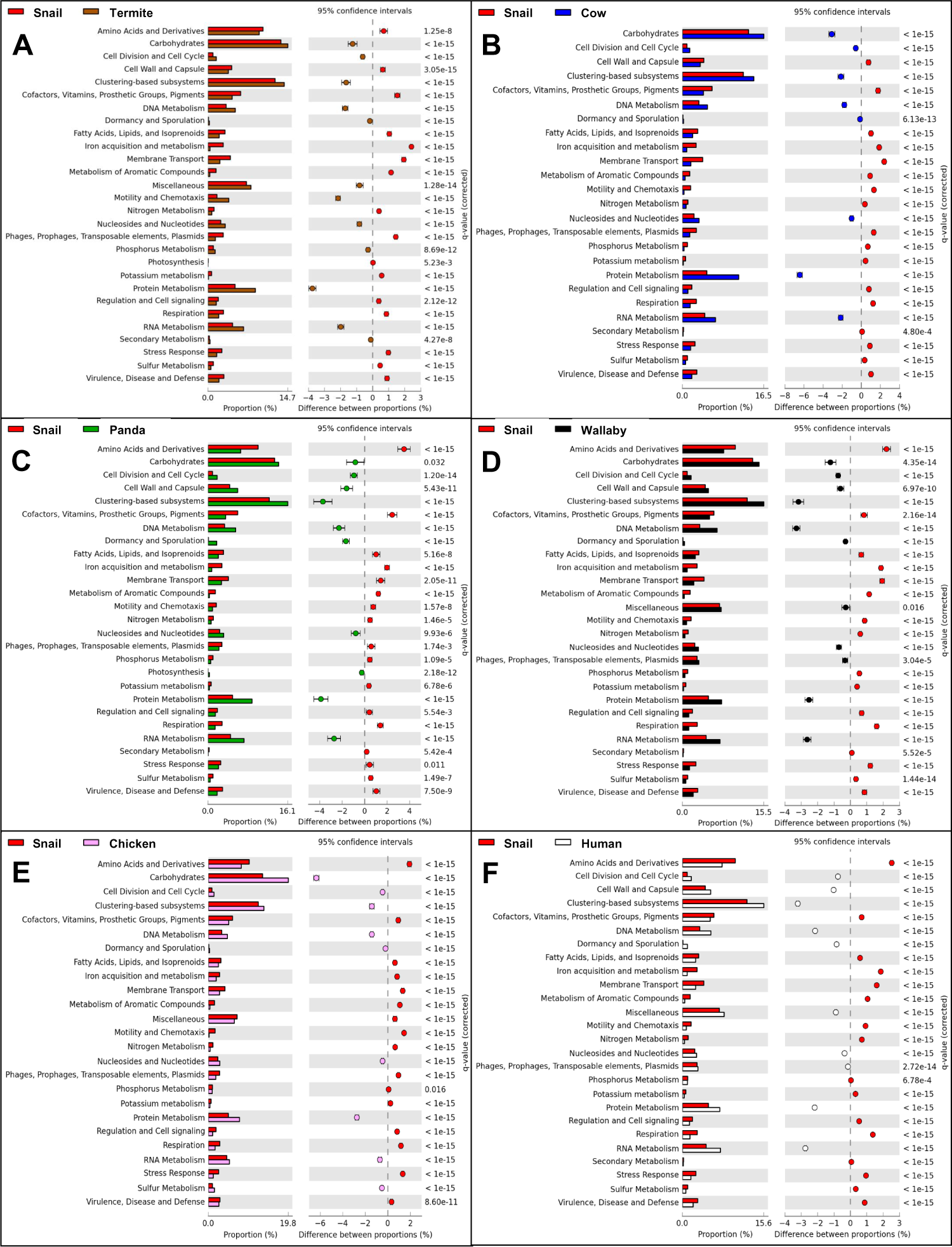

Supplement: Figure S4 — Extended error bar of functional gene groups from Achatina fulica versus other gut metagenomes. Pair-wise comparisons were calculated for the snail metagenome versus (A) termite, (B) cow rumen, (C) panda, (D) wallaby, (E) chicken, and (F) human metagenomes are shown. (TIF) [file pone.0048505.s004.tif]

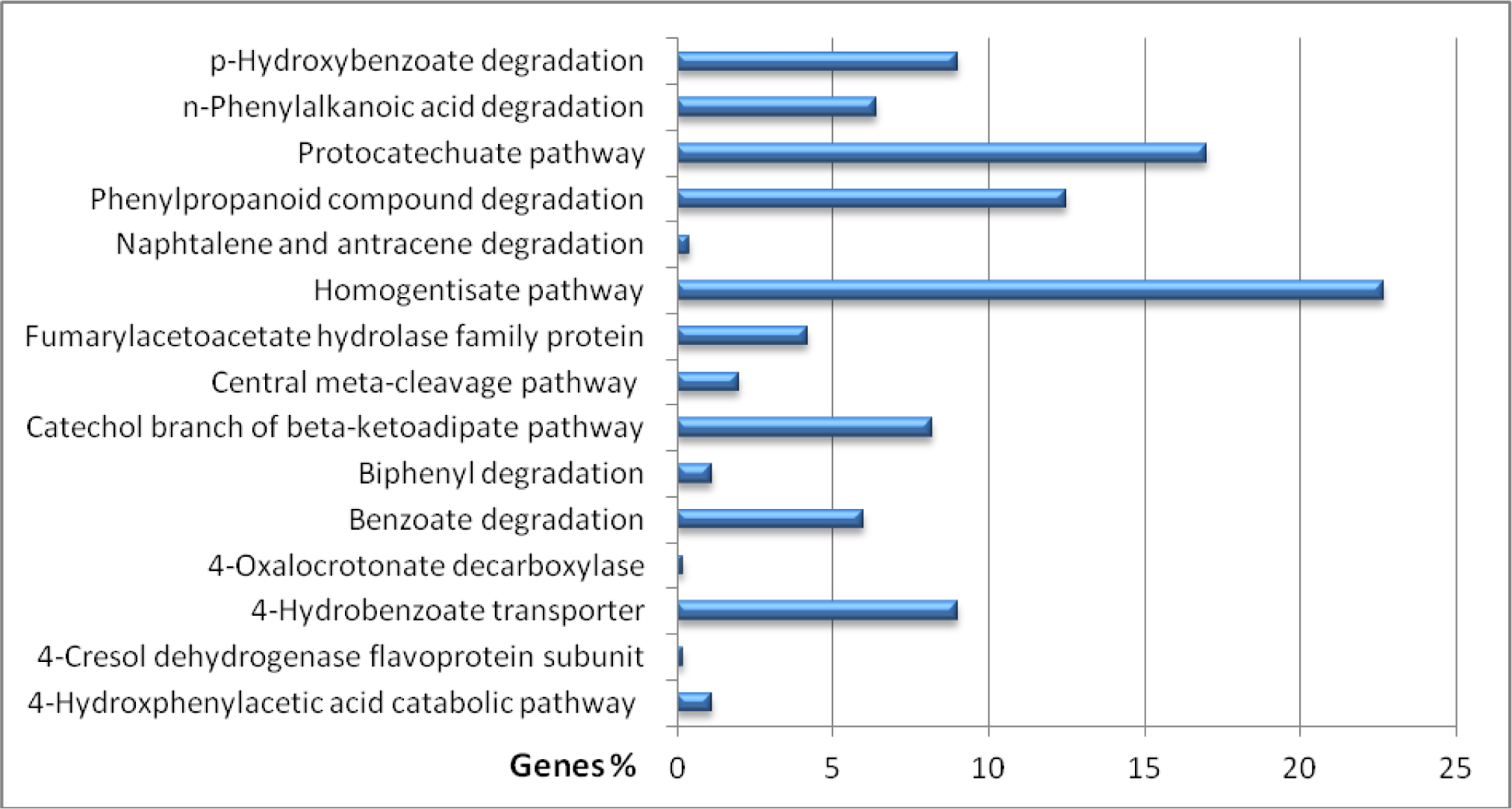

Supplement: Figure S5 — Functional analysis (SEED) for Metabolism of Aromatic Compounds category. (TIF) [file pone.0048505.s005.tif]

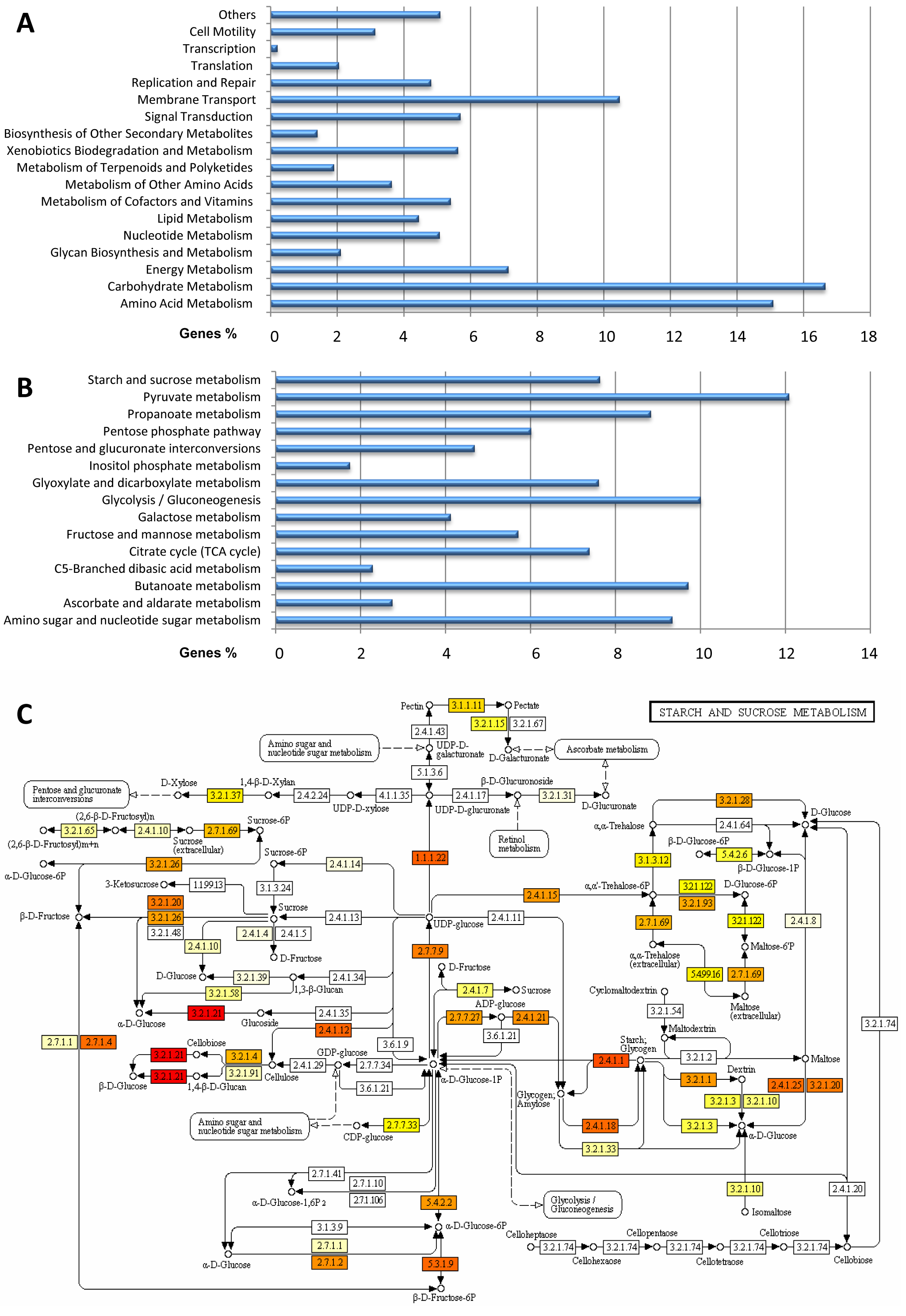

Supplement: Figure S6 — Functional analysis based on the KEGG database. A) Relative number of genes from the snail crop metagenome classified according to the KEGG main categories. As expected, the sequences related to carbohydrate and amino acid metabolism predominate, accounting for 17% and 15% of total coding sequences related to KEGG pathways, respectively. B) Examination of the relative gene diversity within the KEGG pathway for carbohydrate metabolism. C) KEGG sub-pathway for starch and sucrose metabolism. The color scale represents the number of genes (in logarithmic scale) found for each KEGG entry in the giant snail metagenome. Notice that a great variety of putative genes are related to cellulose degradation, including 49 endo-1,4-β-D-glucanases (entry 3.2.1.4), 3 types of 1,4-β-cellobiosidases (3.2.1.91), and 390 β-glucosidases (3.2.1.21). Furthermore, 27 sequences were classified as putative xylan-degrading enzymes (3.2.1.37, 1,4-β-xylosidases). (TIF) [file pone.0048505.s006.tif]

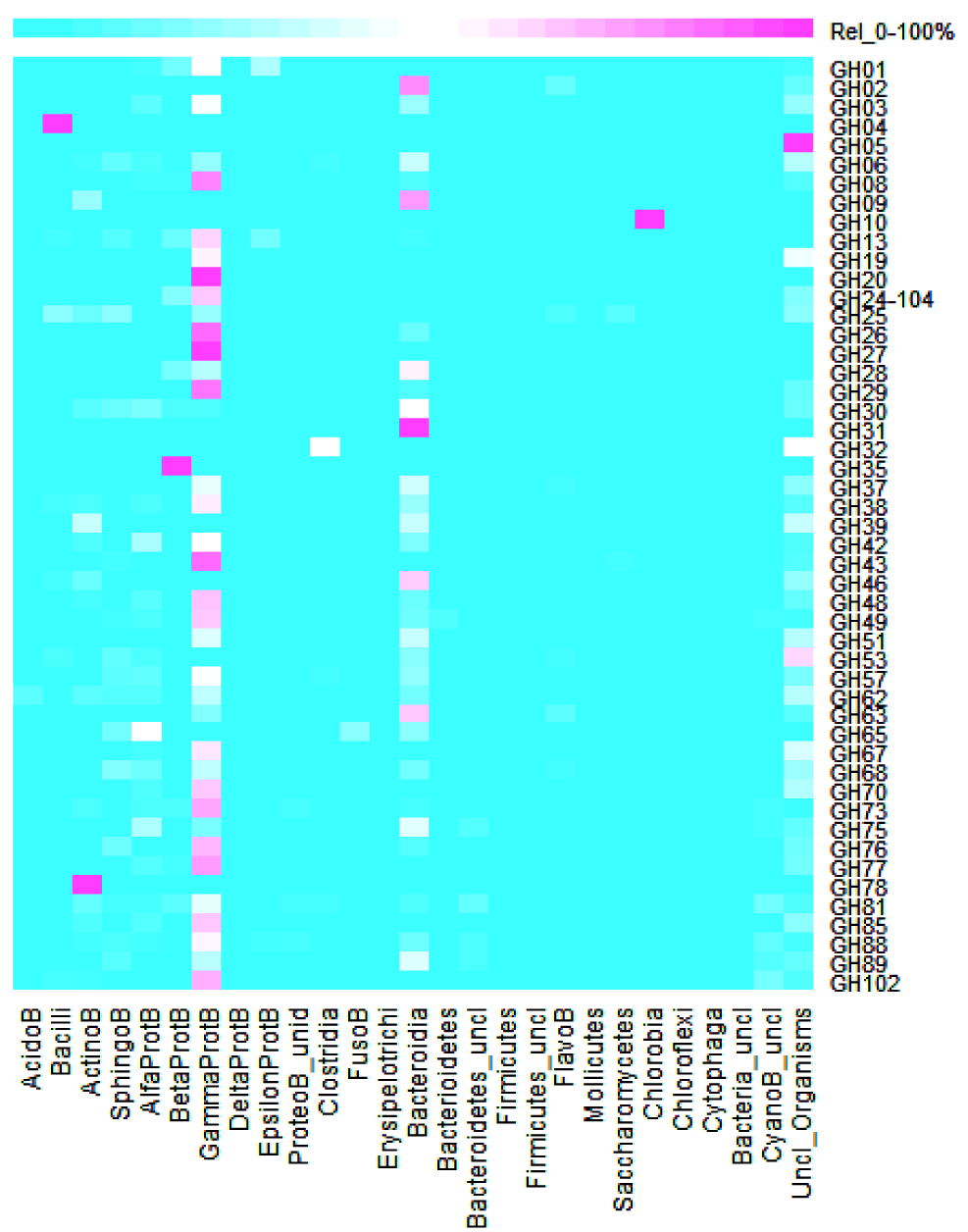

Supplement: Figure S7 — Contribution of organism taxa (Class level) to glycoside hydrolase (GH) hits on Achatina fulica metagenome. The color represents the proportion of each organism shown relative to the number of each GH hit. The scale is linear; red indicates 0, and blue indicates 100%. (TIF) [file pone.0048505.s007.tif]
