# Supplementary material for: Metagenomic Analysis of the Microbiota from the Crop of an Invasive Snail Reveals a Rich Reservoir of Novel Genes
Source: PLoS One. 2012 Nov 1;7(11):e48505. doi: 10.1371/journal.pone.0048505 (PMC3486852; doi:10.1371/journal.pone.0048505)
Supplement: Table S2 — Summary of metagenomic data obtained from the Achatina fulica crop microbiome. (DOC) [file pone.0048505.s009.doc]

**Table S2. Summary of metagenomic data obtained from *Achatina fulica* crop microbiome.**

| **Parameters** | **Filtered** | **Non-filtered** | **Total** | **Assembly** |
| --- | --- | --- | --- | --- |
| **MG-RAST ID**  **No. of sequences** | 4460151  739,381  525,841**^*^** | 4481175  558,217  384,126**^*^** | 4480501  1,297,598  909,967**^*^** | 4482672  426,017**^*^** |
| **Avg. length (bp)** | 347 ± 80**^*^** | 352 ± 82**^*^** | 349 ± 81**^*^** | 370 ± 912**^*^** |
| **Total length (bp)** | 182,716,265 | 135,384,210 | 318,100,475 | 158,000,172 |
| **Predicted proteins^†^**  **No. of rDNA hits^‡^**  Bacteria (R/S/G)  Archaea (R/S/G)  Eukarya (R/S/G)  Unassigned (R/S/G)  **LCA^¥^**  Bacteria (%)  Archaea (%)  Eukarya (%)  Viruses (%)  Unclassified (%)  No hits (%) | 281,690  (975/982/798)  (0/0/0)  (0/2/0)  (151/304/264)  463,975 (88.24)  63 (0.012)  549 (0.104)  473 (0.090)  39 (0.007)  60,742 (11.55) | 219,893  (479/415/322)  (0/0/0)  (0/16/0)  (44/183/164)  261,779 (68.15)  24 (0.006)  1,897 (0.494)  11,571 (3.012)  31 (0.008)  108,824 (28.33) | 473,379  (1498/1389/1141)  (0/0/0)  (0/18/0)  (173/466/391)  729,300 (80.15)  88 (0.010)  2,443 (0.268)  12,123 (1.332)  73 (0.008)  165,940 (18.24) | 294,404  (81/71/58)  (0/0/0)  (0/4/0)  (9/35/23)  347,291 (81.52)  44 (0.010)  1,656 (0.389)  1,624 (0.381)  43 (0.010)  75,359 (17.69) |

**^*^**After duplicate removal, splitting and trimming of sequence reads.

^†^Predicted protein coding regions assigned an annotation using at least one of protein databases (M5NR) in MG-RAST server.

**^‡^**The evalue cutoff for rDNA hits for all databases used (R: RDP; S: SILVA; G: Greengenes SSU) is 1e-5 with a minimum alignment length of 50 bp.

**^¥^**Lowest common ancestor (LCA) using 1e-5 cutoff.
